# A Study of Ten Cases of Operation for Perforated Gastric Ulcer

**Published:** 1908-09

**Authors:** Charles A. Morton

**Affiliations:** Professor of Surgery in University College, Bristol; Senior Surgeon to the Bristol General Hospital and the Royal Hospital for Sick Children and Women; Consulting Surgeon to the Cossham Memorial Hospital


### A STUDY OF TEN CASES OF OPERATION FOR PERFORATED GASTRIC ULCER.

BY

CHARLES A. MORTON, F.R.C.S.,

*Professor of Surgery in University College, Bristol; Senior Surgeon to the Bristol General Hospital and the Royal Hospital for Sick Children and Women; Consulting Surgeon to the Cossham Memorial Hospital.*

---

I FIND, on looking up my records, that I have operated on ten cases of perforated gastric ulcer, with seven recoveries, and I propose in this paper to give a brief abstract of them,[1] and to point out the lessons I have learnt from them.

As we might expect, the chance of recovery depends very largely on two things—one, the time which elapses between perforation and operation, and, the other, the extent of extravasation of stomach contents. The following table by Robson and Moynihan[2] shows the relation of the result to time of operation :—

| | Total Cases. | Recovered. | Died. | Mortality. |
|---|---|---|---|---|
| Operation under 12 hours . . | 49 | 35 | 14 | 28.5 |
| ,, from 12—24 hours | 33 | 12 | 21 | 63.6 |
| ,, from 24—36 hours | 16 | 2 | 14 | 87.5 |
| ,, from 36—48 hours | 2 | 0 | 2 | 100.0 |
| ,, over 48 hours . . | 33 | 16 | 18 | 51.5 |

But it seems uncertain whether these statistics are taken from consecutive series of cases. If not, they will almost certainly fail to give the real mortality, for single fatal cases do not as a rule get published. Paterson found that in a series of consecutive cases[3] operated on within twelve hours the mortality was 47 per

[1] It is a curious fact that I had been Surgeon to the Bristol General Hospital for seven years before I operated on my first case of perforated gastric ulcer.

[2] *Diseases of the Stomach*, 1901, p. 161.

[3] *Lancet*, 1906, i. 575.

cent., within twelve to twenty-four hours 50 per cent., and from twenty-four to forty-eight hours 83 per cent. The difference between his first and second periods of twenty-four hours is therefore very marked.

All my three fatal cases had general peritonitis. In one thirty-nine hours had elapsed between perforation and operation, in another forty-nine, and in the third three days; but one of the successful cases (Case 3) was not operated on for thirty-nine hours after perforation, but in this case the extravasation was slight, and the peritonitis localised. In two of these three cases the patient was in a very collapsed state before operation, and the third died nearly three months after operation, and some time after a subphrenic abscess had been drained.

The mortality after operation for perforated gastric ulcer has been considerably reduced in the last few years, owing perhaps to earlier diagnosis. Mr. Crisp English[1] found that only 52 per cent. recovered out of forty-two consecutive cases at St. George's Hospital up to 1903; and Mr. Sargent[2] gives 58 per cent. recoveries in forty-nine cases at St. Thomas's Hospital up to 1904. Mr. Moynihan's results, published in 1906,[3] are much better—66.6 per cent. recoveries in twenty-seven cases; and Mr. Crisp English and Mr. Turner together, at St. George's Hospital, had nine cases with only one death. In my ten cases the recoveries were 70 per cent.

*Diagnosis.*—The diagnosis of several of my cases was not difficult, but it is worth while calling attention to what has been called the "latent period." The symptoms of onset may be most acute, and then in a few hours there may be so little pain that you are put off your first diagnosis. In Case 4 the diagnosis was much less certain when we saw the patient just before operating than it seemed earlier in the day, but fortunately we decided to operate. In other cases in this series it will be noted that the severe pain had passed away before I saw the patient. But in the latent period the rigidity will usually persist, and also a certain

[1] *Med.-Chir. Trans.*, 1904, lxxxvii. 27.

[2] *St. Thomas's Hosp. Rep.*, 1904, xxxiii. 477.

[3] *Med.-Chir. Trans.*, 1906, lxxxix. 471.

amount of tenderness, though the frequency of the pulse may lessen.

In two of my cases appendicitis was diagnosed (Cases 5 and 7). In Case 5 the tenderness and fulness were most marked in the appendix region, and in Case 7 the history was that the pain had been there. This is not at all an uncommon mistake, especially with perforating duodenal ulcers—indeed, with the latter it is quite frequent. Extravasated fluid runs down into the right iliac region in these cases, and peritonitis is marked there. The mistake matters really very little, provided we operate, for we can easily extend our incision in the rectus up to the stomach region; but the fact that in nineteen out of fifty-one cases of perforated duodenal ulcer collected by Moynihan a diagnosis of appendicitis was made, is an additional argument in favour of early operation in all cases diagnosed as acute appendicitis with severe symptoms at the onset, at any rate in adults.

It seems to me that rigidity of the abdominal wall is one of the most important signs of perforated gastric ulcer, though when general peritonitis was present in Case 8 there was neither rigidity nor tenderness, but the patient was in a very collapsed condition.

In connection with the diagnosis of perforated gastric ulcer, it may be well to call attention to a source of error pointed out by Moynihan.[1] He says he knows of three cases in which negative exploration for perforated gastric ulcer was performed in women at the commencement of menstruation. Sharp abdominal pain, with vomiting, distension, and collapse were the symptoms and signs present. In one of his own cases (in which he did not explore) the absence of any rigidity and tenderness in the abdomen negatived perforation. There may be a history of previous similar attacks at menstrual times.

We must not fail to diagnose a case as one of perforated gastric ulcer because shortly after the onset of symptoms shock is not present. In one of my cases (Case 9) I saw the patient one hour after perforation; his pulse was 112, not very bad, nor did his general condition suggest collapse. Neither was Case 1, see a few hours after perforation, suffering from collapse. Moynihan

[1] *Abdominal Operations*, 1906, p. 144.

says[1] that within the first two hours, though the pain is very severe, there is no rigidity or collapse, or any marked rise in pulse rate. However, in one case I saw an hour after perforation the whole abdomen was very rigid, and I believe collapse may be very marked. Mr. Pearce Gould[2] refers to a case of sudden death from the shock of perforated gastric ulcer.

*Treatment.*—The method adopted for cleansing the peritoneal cavity differed in the successful cases—in some I sponged, and in others I flushed. If the extravasation and effusion of fluid and lymph is localised, it seems to me that it is unwise to risk further dissemination by flushing. In cases in which extravasation or inflammatory effusion is widespread, especially if stomach contents are widely diffused, perhaps flushing is the more satisfactory method, as there is less manipulation, and therefore less tendency to shock, and I should expect the cleansing to be rather more thorough than with sponging, if plenty of saline solution is used;[3] but I have learnt from my experience of the treatment of diffuse peritonitis from appendix disease how well most cases do after sponging, and unless there were evidence of very wide—almost general—diffusion of infective material, there is a risk of infecting fresh areas in flushing. Paterson[4] is opposed to irrigation, because he has observed when giving the anæsthetic that if the patient was not very deeply under, when flushing was used there was weakening and quickening of the pulse. But he does not tell us if he has observed the effect of sponging on the pulse. He only recommends sponging of the space "above and behind the liver." He suggests sucking out the fluid from the pelvis. He avoids sponging, because he thinks it will interfere with peristalsis after operation, and he seems to depend more on the action of purgatives after operation to cleanse the peritoneal cavity than on flushing or sponging at the time of operation. I should rather still be inclined myself to act on advice given by Pearce Gould more than ten years ago—not only to operate early, but to spare no pains in thoroughly cleansing the peritoneal cavity.

1 *Brit. M. J.*, 1906, ii. 1397. 2 *Ibid.*, 1894, ii. 860.

3 I see this line of practice is recommended by Mayo Robson. *Brit. M. J.*, 1906, ii. 1347.

4 *Loc. cit.*

If the intestines have to be turned out of the abdomen in order to get room to flush, then I think there would be greater shock with flushing than with sponging ; but then sponging without evisceration would probably not be effectual in cleansing the peritoneum because of the tight packing of the intestines.

In all the eight successful cases of Crisp English and Turner[1] irrigation was employed, and supra-pubic and loin counter-openings made before the irrigation was started, so that free exit for the fluid was provided. In nearly all the cases the supra-pubic openings disclosed a pelvic collection of "peritoneal fluid and stomach contents." This shows how important it is in all cases of perforated gastric ulcer to examine the pelvis, if not by a separate opening, at any rate by inserting into it a sponge on a holder. If fluid is found, then I should certainly make a supra-pubic opening for cleansing and drainage, but I should not do so in every case, nor make counter-openings in the loins either. The right kidney pouch and the left loin can both be drained by long split tubes with gauze wicks from the anterior abdominal wound, but in some cases of very extensive infection of one or other loin no doubt a counter opening there would be advisable. In every case, even though the extravasation or the peritonitis was quite localised around the perforation, I should certainly drain, and of all drains the split drainage tube with gauze wick seems to me the best.

Barker[2] has recorded twelve consecutive cases of perforated gastric ulcer treated by sponging, with five recoveries. If we compare the results with Crisp English and Turner's after irrigation, we must remember that so much depends not only on the period which elapsed between the perforation and the operation, but on the extent of extravasation of stomach contents, that we can hardly conclude that irrigation is the best method to employ in all cases.

In all my cases but one I have been able to close the perforation by suturing, though in some it was very difficult to get the stitches to hold in the indurated friable tissue around the perforation, and in several the suturing was supplemented by an omental

[1] *Loc. cit.* [2] *Tr. Clin. Soc. Lond.*, 1900, xxxiii 39.

graft. In Case 4 I could not suture, the induration around the perforation was so great, but omental grafting alone answered well.

It is a mistake to suppose that free gas is always present in perforated gastric ulcer. It was suggested some years ago that all that was necessary to settle the diagnosis of doubtful cases of perforated gastric ulcer was to make a very small incision and see if free gas was present. If this test had been relied on, many perforations would have been missed. You must examine every part of the stomach. If you find no perforation anteriorly, you must divide the gastro-colic omentum, and examine the posterior surface of the stomach, though perforations here are rare. All my ten cases were anterior perforations. You are often led to the perforation by increased redness and lymph deposit on the wall of the stomach, and by pressure on it gas and stomach contents may be forced out. It has been suggested that air may be pumped through the perforation through an œsophageal tube passed from the mouth, but pressure on the stomach ought to be sufficient to cause the exit of gas or fluid at the perforation.

Because free gas is often not present in the peritoneal cavity in these cases, there may be no alteration in the liver dulness. I should say there rarely is obliteration of it, even if free gas is present. In a distended abdomen it may be obliterated from distension of the intestines, but in a flat abdomen I have seen it absent and no free gas present, so that I do not regard the presence or absence of liver dulness as a very reliable guide in these cases. Barker[1] has come to much the same conclusion from a study of his twelve cases. He found liver dulness present with abundant free gas, and absent without any free gas.

I have never felt inclined to adopt the suggestion made of late years by some surgeons, to do gastro-jejunostomy as well as close the perforation. Patients with perforated gastric ulcer do not seem to me suitable cases for any more operative interference than is absolutely necessary to save life, even in cases of early operation, and they do very well with suture alone if peritonitis has not already set in. Nor, for the same reason, should I excise a per-

[1] *Loc. cit.*

forated gastric ulcer, though several surgeons have done this, but not with much success.[1]

Mayo Robson says[2] that if the patient's condition will permit of it, the question as to the performance of gastro-jejunostomy should be considered, because other ulcers may be present and will be cured, and if on the point of perforating, perforation will be prevented, the sutured ulcer will heal better, there will be less risk of hæmatemesis, saline purgatives can be given and will assist in the absorption of peritoneal fluids, and early mouth feeding can be resorted to. Paterson[3] recommends it for much the same reasons, and Moynihan[4] seems inclined to recommend it in early operations, to aid the healing of the sutured ulcer, and to cure others which he says are usually present. As he points out, if in closing the perforation we have to seriously narrow the pyloric part of the stomach, it may be absolutely necessary, but the other reasons seem to me hardly urgent enough to demand it, for even though the operation is done early and the patient may bear the earlier stage of the operation well, yet we must remember that there has been very severe pain, and the less manipulation of abdominal viscera the better.

It may be necessary to do gastro-jejunostomy later for the condition of the stomach resulting from the ulcer, or if the ulcer will not heal with prolonged medical treatment. In one of my cases (Case 4) I had to do the operation for "hour-glass" stomach, due to contraction of a chronic ulcer six months after the closure of the perforation. But because gastro-jejunostomy may ultimately be necessary, I should not do it at the time of closure of the perforation. Some surgeons would, however, regard the possibility of later trouble as an argument for the performance of gastro-jejunostomy at the time of closure of the perforation.

**Case 1.**—Mrs. M., 40., March, 1908. Children's and Women's Hospital. History of pain and vomiting after food for some months. Pain very severe below left costal margin, extending up chest into left arm. Came on three hours after dinner. Four hours later seen with Dr. Osmond Bodman. Not then in much

[1] Paterson, *loc. cit.*

[2] *Brit. M. J.*, 1906, ii. 1347.

[3] *Loc. cit.* [4] *Abdominal Operations*, 1906, p. 148.

pain, but could not breathe deeply. Pulse under 100. Abdomen tender and rigid almost all over, but especially just below left costal margin. Three and a half hours later tenderness and rigidity were much less, but still marked below the left costal margin. She seemed easy, and could breathe more deeply. Operation eight hours after perforation. Perforation in anterior wall, near œsophageal orifice, with free escape of gastric fluid but no free gas. Brown fluid in pelvis and lymph in both renal regions as well as in region of stomach. Perforation sutured. Infected parts thoroughly sponged. Supra-pubic drainage opening, and drainage tube into right loin and splenic region from original wound. No shock. She made a good recovery. Feeding by nutrients only for fourteen days, and then by liquid only for some time

**Case 2.**—Miss B., 25, January, 1907. General Hospital. At onset very severe epigastric pain, four hours after a light meal, and continued for hours until opium given. I saw her with Dr. Cook thirty-five hours after perforation. There was then very little pain, and there had been no vomiting save a little retching at onset of the pain. There was a history of gastric ulcer five years ago, and a little epigastric pain a few days before. Considerable general tympanitic distension was present, with tenderness and rigidity under the left costal margin. Pulse 140; temperature 102°. Operation thirty-seven and a half hours after perforation. Perforation on anterior surface, just below lesser curve, and three inches from pylorus. Sutured, and omental graft used. Lymph and serous fluid in upper abdomen, including right kidney pouch, none in lower. Sponged. No counter openings, but many drainage tubes with wicks. Moderate shock. Fed by nutrients only for some time, then liquids only. Twenty-four days after operation began to expectorate pus, and serous fluid found at left base. Both conditions cleared up. Discharged quite well two months after operation.

**Case 3.**—Rose G., 20, November, 1906. General Hospital. Epigastric pain after food fourteen days. Onset 6.30 a.m. before any food taken, and very severe pain in epigastrum. I saw her thirty-six hours after perforation (had taken milk in meantime), and then she was not in pain. Moderate general distension, with slight general tenderness, but marked tenderness and rigidity over upper left rectus. Condition essentially the same three hours later. Pulse 120. Operation thirty-nine hours after perforation Upper left rectus remained rigid even under anæsthetic. Localised peritonitis about stomach. Perforation in lesser curve near œsophagus. Sutured. Pulse 140, but not feeble at end. Fed by nutrients only for fourteen days. Discharged well about six weeks after operation.

**Case 4.**—Miss R., 45, April, 1905. Gastric pain and vomiting for one month, and same symptoms with hæmatemesis some years before. At onset very severe abdominal pain, so that she could not move, and felt " as if something had broken in her side." Dr. Michell Clarke saw her seven and a half hours after perforation, and I saw her with him shortly after. Pain was then severe in epigastrum and left loin anteriorly, and she could not breathe deeply enough to speak plainly (as in Case 1). Pulse 120, not feeble. No vomiting. Twelve hours after perforation, she was quite free from pain (no morphine) and pulse was less rapid, but the upper abdomen, especially the upper part of the left rectus, was very rigid and very tender. Operation thirteen and a half hours after perforation. Perforation in lesser curve. There was very great induration around, so that it was not possible to suture. The perforation was closed by an omental graft. There was only localised extravasation of stomach contents. Sponging and free drainage. No serious shock.

**Case 5.**—K.M. (female), 19, March, 1905. General Hospital. Suffered from epigastric pain and nausea for some time. At onset very severe pain, " doubling her up," and vomiting. Pain and vomiting continued. Seen by me about forty-six hours after perforation. Pulse 140, feeble; temperature 100.6°. Tense general abdominal distension and tenderness, most in right lower abdomen, but no rigidity. The greatest tenderness and fulness being in the right lower abdomen, I opened the abdomen here forty-nine hours after perforation, thinking it might be appendix trouble. I found a diffuse purulent peritonitis. Perforation was near the pylorus, and covered by lymph. Lymph peeled off, and perforation sutured. Sponged and counter opening for drainage made above pubes. Multiple drains. By fourth day much better. Pulse 104. Vomiting ceased. Abdomen hardly at all distended, soft, and not tender. Mouth feeding (peptonised milk) started. A large left subphrenic abscess developed and was drained. Intractable diarrhæa and epileptiform convulsions set in, and she died nearly three months after operation. At *post-mortem*, perforation sutured at operation found as a scar, and another ulcer had perforated, but was closed by adhesions. An abscess was found in the liver, and a considerable quantity of serous fluid with flakes of lymph in the pelvis, and a little pus was shut up amongst adherent coils of intestine.

**Case 6.**—Mabel R., 14, November, 1904. Children's and Women's Hospital. Epigastric pain and vomiting for a week. Then severe sharp pains which persisted, but she walked from city up to hospital the next morning, after a light breakfast, when the pain got more intense, and she became collapsed. It is not easy in this case from the symptoms to fix the time of perforation, but from the condition within the abdomen I should say it took place that morning, and that the other symptoms were due to peritonitis at the base of the ulcer. I saw her about two hours after the most intense pain came on. The abdomen was board-like all over, and tender below the left costal margin, and in left iliac fossa, and hardly at all distended. The severe pain had gone, and she only had pain on breathing deeply or moving. Operation four hours later. Then temperature 102°. Perforation on anterior surface near pylorus was found and sutured. Clear fluid in left iliac fossa and pelvis. Irrigated with counter opening for drainage above pubes. Lymph on stomach, but no general peritonitis. No shock. Discharged well a month after operation. No complications.

**Case 7.**—E.C. (female), 25, May, 1908. At onset severe abdominal pain. Seen with Dr. Eberle and Dr. Walker Dunbar thirty-six hours after perforation. Another severe attack of pain ten and a half hours before I saw her. Pain had been on right side of abdomen, and ruptured appendix abscess was diagnosed. Temperature had been 102° earlier in the day. Pulse too feeble to count, and she seemed too bad to have much chance of recovery, but her relations wished to give her any slight chance which operation might give, if she died under it. Abdomen was much distended, rigid, and generally tender. Operation thirty-nine hours after perforation. Perforation near pylorus on anterior surface, with general peritonitis. Flushed. Died at end of operation.

**Case 8.**—Male, 18, May, 1903. General Hospital. Onset with severe pain in left abdomen. Supposed to be suffering from pleurisy before admission. Friction heard probably peritoneal. Admitted three days after perforation, and when seen by me a few hours after admission pulse was very feeble (130) and extremities chilly, though a warm evening. Abdomen distended with shifting dulness, but no tenderness or rigidity. Immediate operation. Perforation on anterior surface near pylorus. Sutured. General peritonitis. Flushed. Died soon after.

**Case 9.**—Mr. V., 45, July, 1903. General Hospital. Had been medical out-patient for gastric symptoms before onset of severe general abdominal pain and vomiting, when lifting a heavy weight. Seen an hour after. Then in great pain all over abdomen, and abdomen very rigid and tender all over. Loud friction heard over lower part of left chest below nipple. Operation five hours after perforation. Perforation on anterior surface close to pylorus. Sutured. Fluid widely extravasated. Flushed. No shock. Fed by nutrients only for ten days, and then only liquids for a time. Discharged well about two months after operation. Some bronchitis the only complication.

**Case 10.**—E.M.W. (female), 26, October, 1901. General Hospital. For three months subject to epigastric pain. At onset severe pain below left costal margin, ten and a half hours before I saw her, when laughing, and two hours after taking a teacup of arrowroot. Had had two previous severe attacks of pain, two days and four days earlier. When I saw her she was not in any pain, but had taken a pill to relieve the pain (prescribed by her doctor) four hours earlier. Abdomen was rigid all over, and very tender below left costal margin. Operation eleven hours after perforation. Perforation near œsophageal orifice on anterior surface, and very difficult to reach. Only localised extravasation. Sponging.

---

